# Supplementary figures and images for: Falcon gut microbiota is shaped by diet and enriched in Salmonella
Source: PLoS One. 2024 Jan 30;19(1):e0293895. doi: 10.1371/journal.pone.0293895 (PMC10826950; doi:10.1371/journal.pone.0293895)

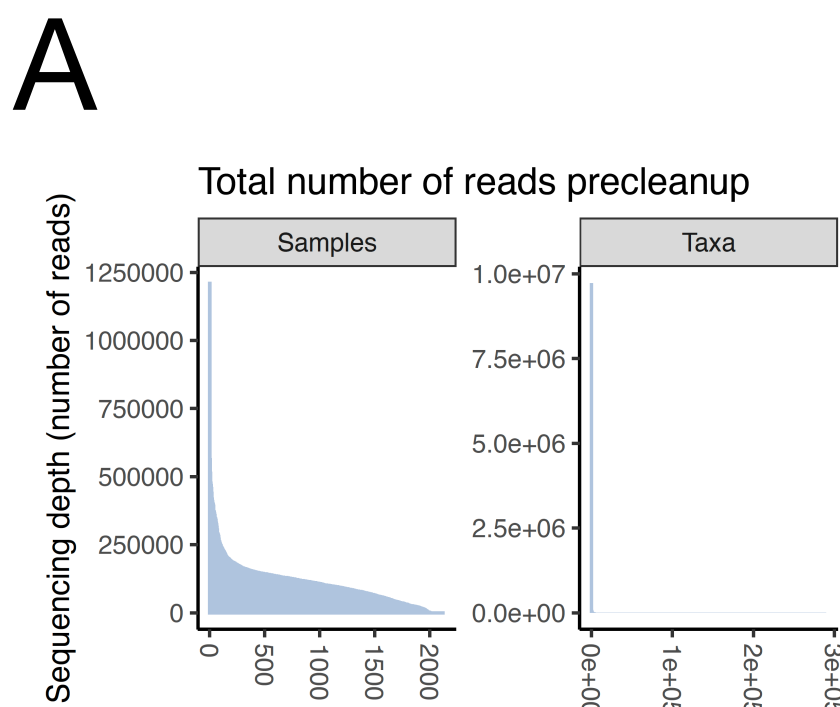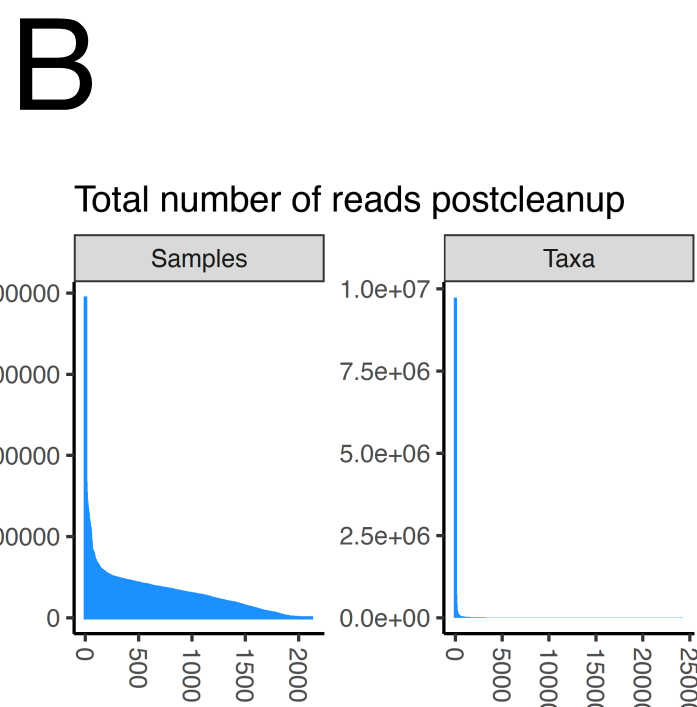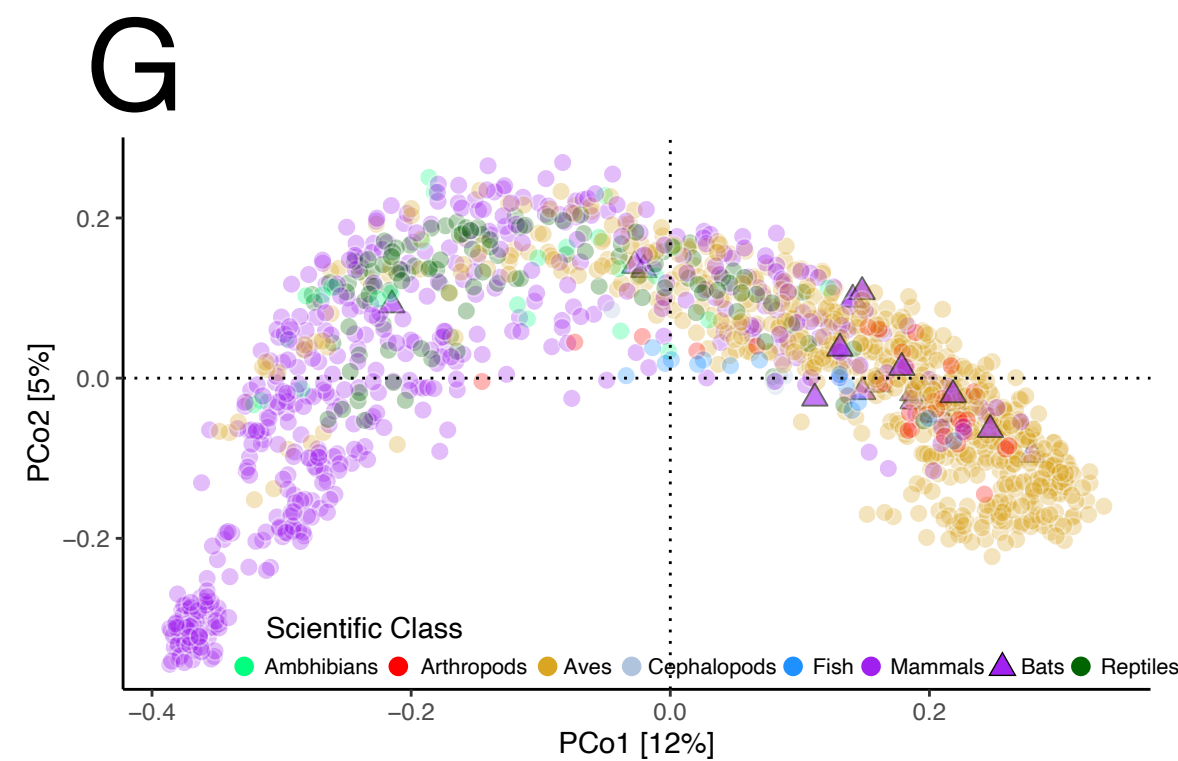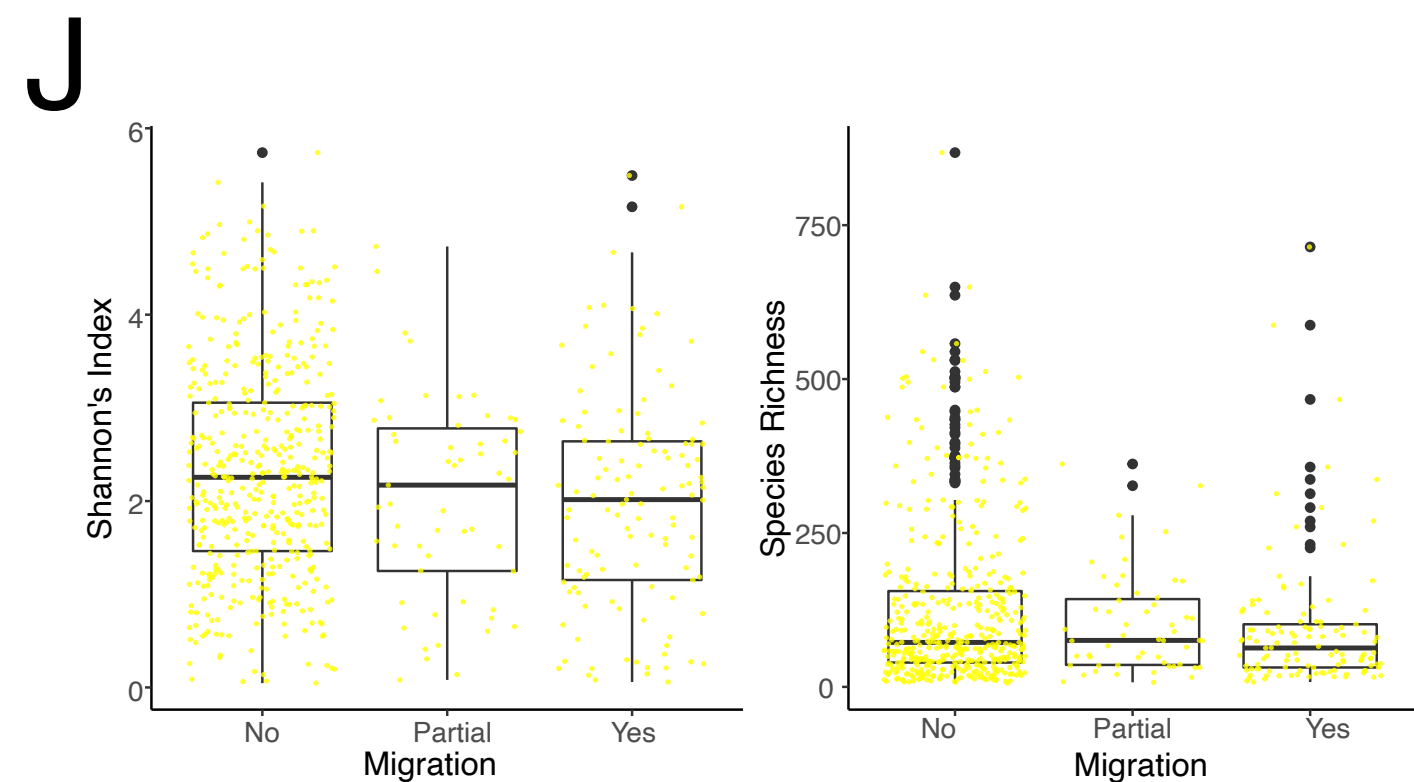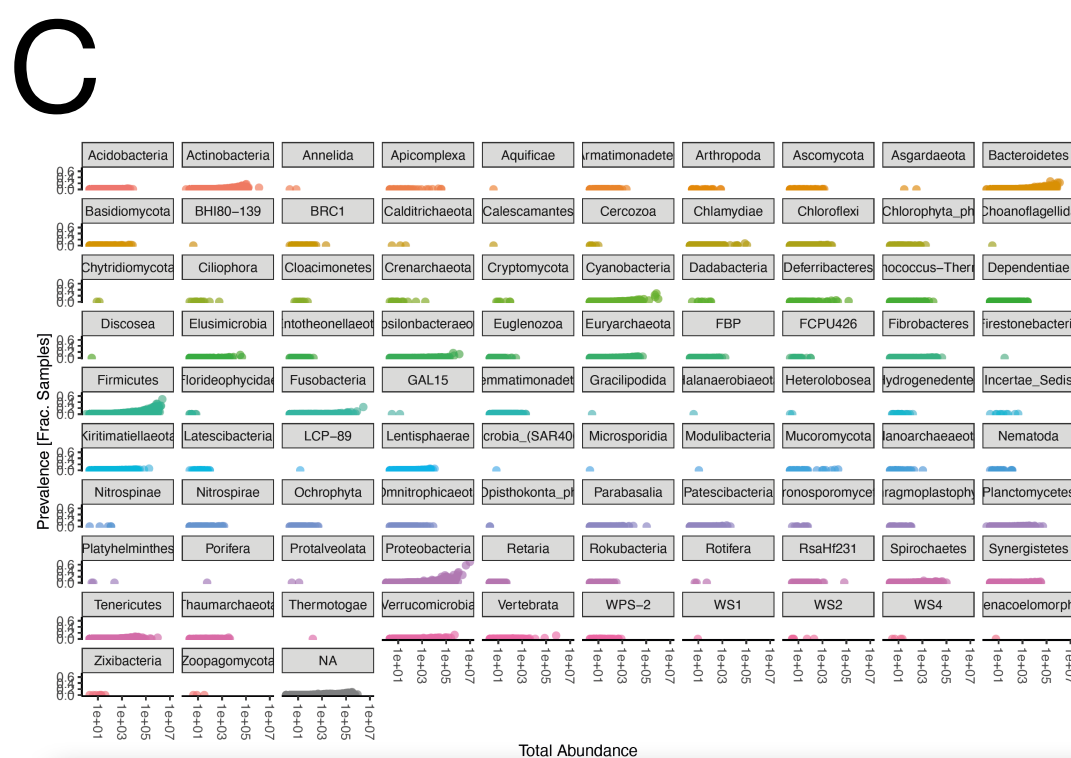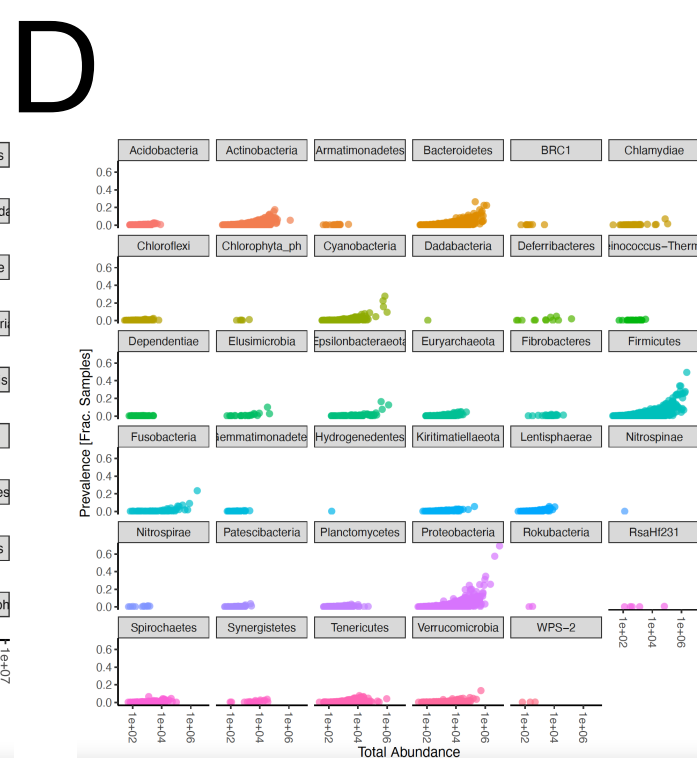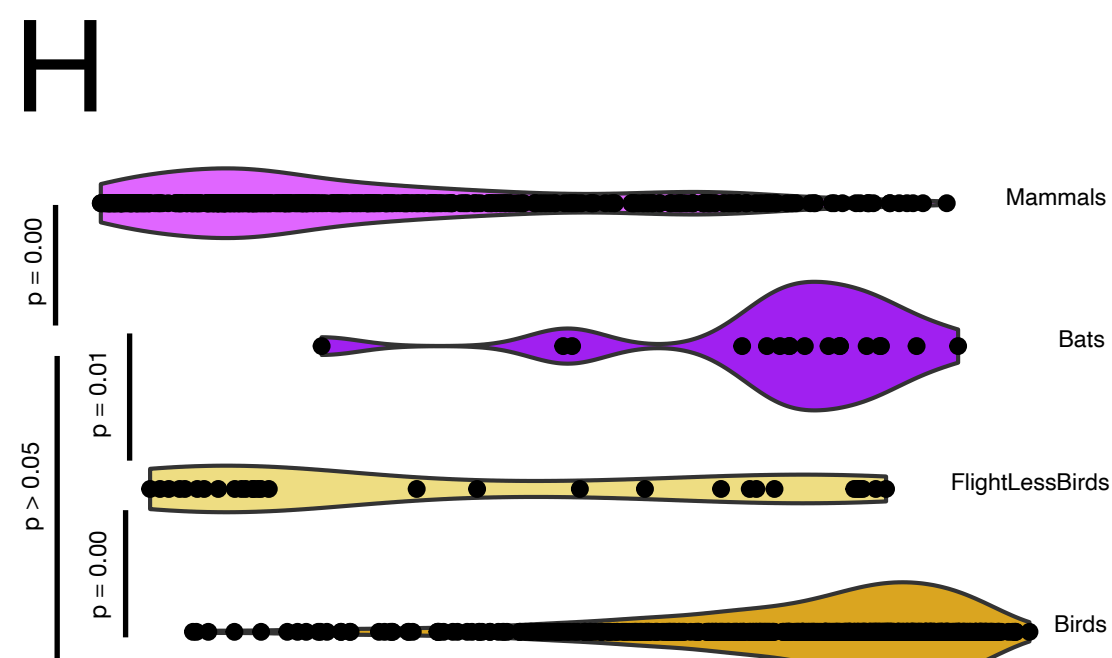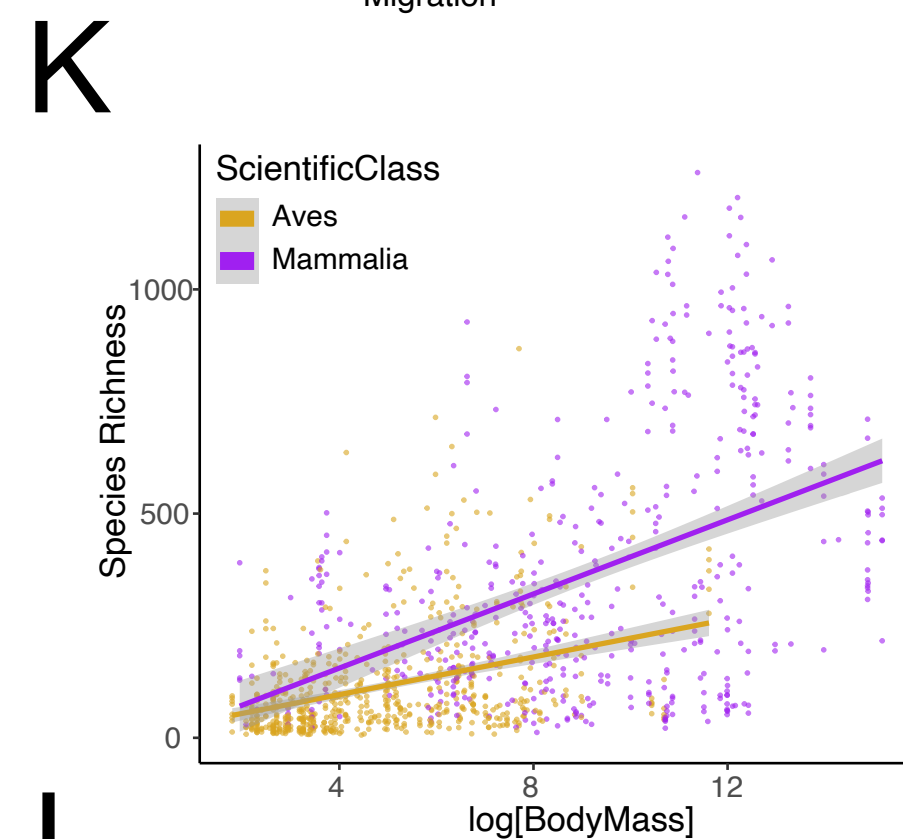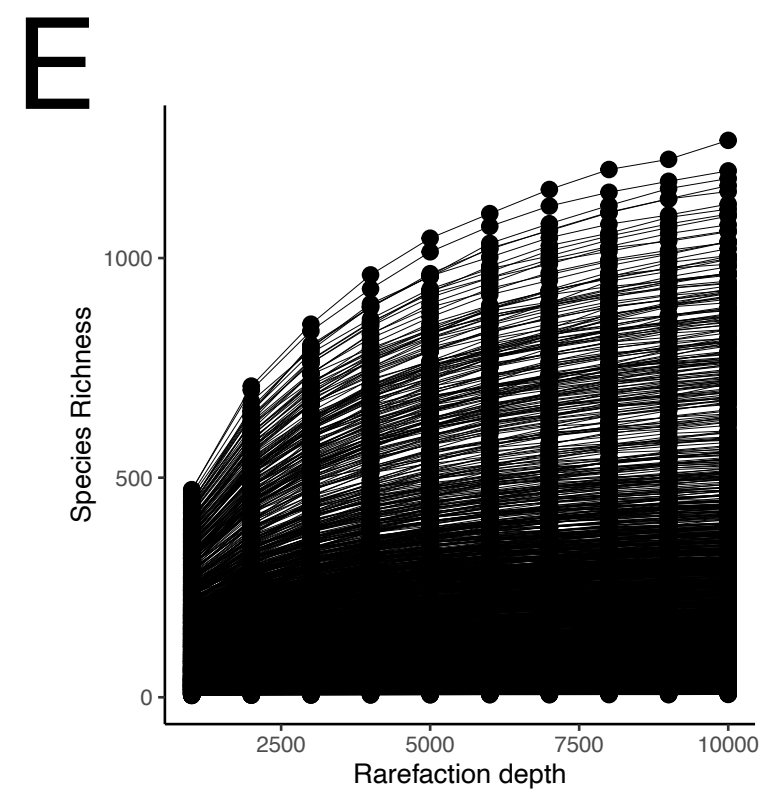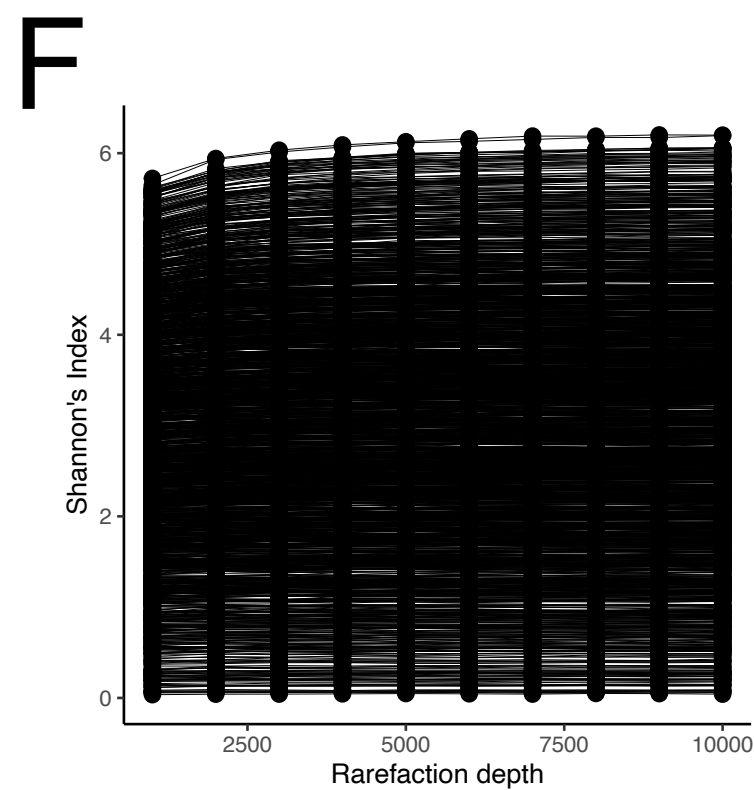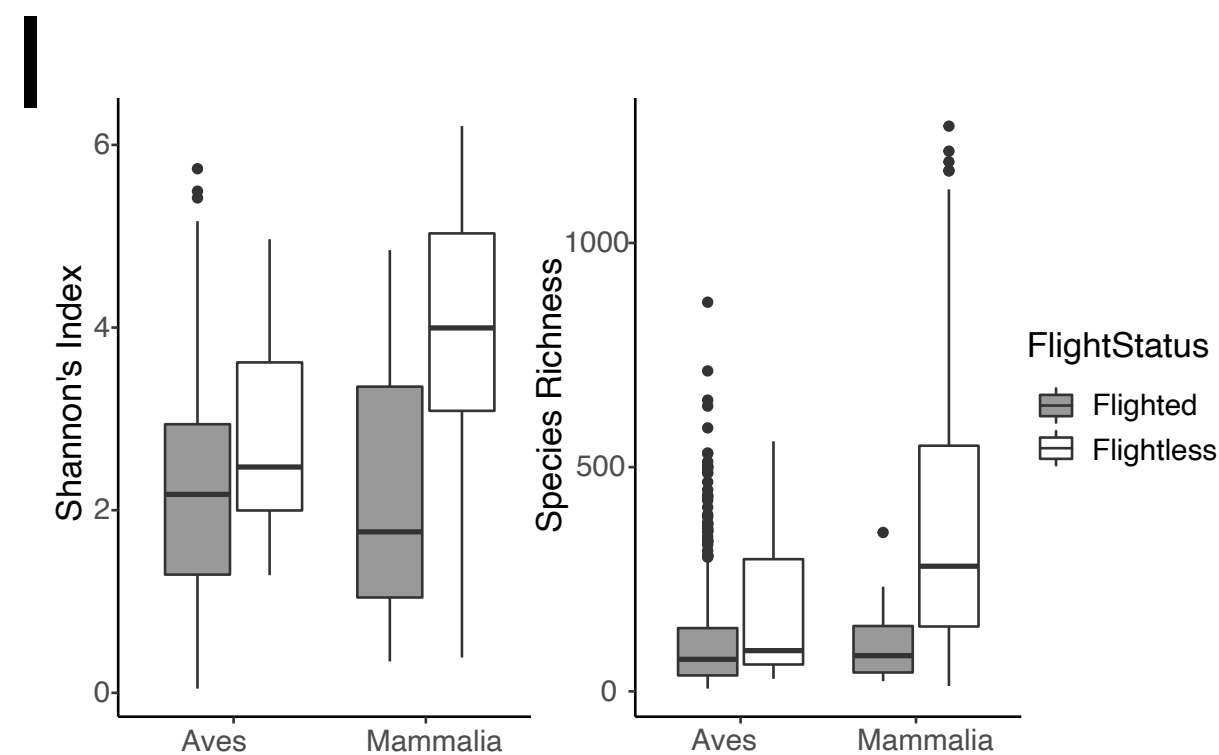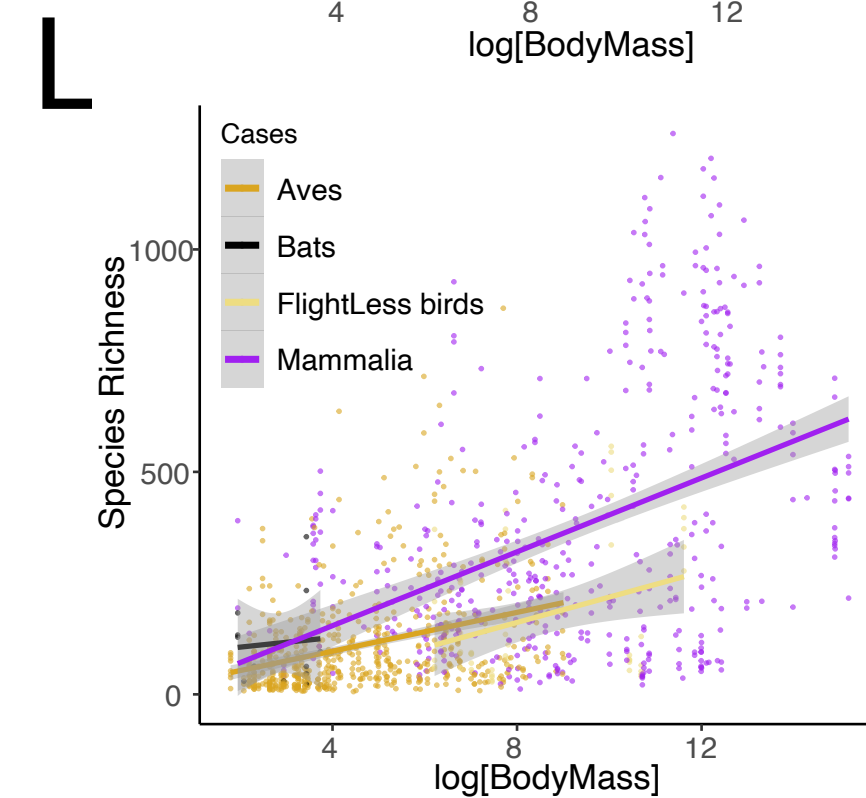

Supplement: S1 Fig — Sequencing depth for each sample and each taxon (A) before filtering any reads, and (B) after removal of lowly abundant ASVs; this does not reduce sequencing depth of either the samples or the taxa significantly. (C) Abundance of Phyla in the dataset. Fungal and eukaryotic phyla were removed from the dataset. (D) After filtering, 35 phyla and a total of 24,000 taxa remained. Rarefaction curves for (E) species richness and (F) Shannon diversity show plateauing alpha diversity for most samples starting at a rarefaction depth of 10,000 reads. (G) Principal coordinate analysis using unweighted UniFrac distances at the ASV level and randomly sampled to include a maximum of 5 individuals per species (1,330 points). Non-flighted mammals (purple dots) have a microbiota distinct from birds (yellow circles) and bats (purple boxes) cluster with birds. (H) Violin plot of PCo1 shows bird and bat microbiota are not significantly different (p = 0.238, Dunn’s post hoc test). (I) Shannon diversity and species richness for flighted and non-flighted birds and mammals. Shannon’s diversity for all pairwise comparisons had p < 0.05, except for flighted mammals vs flighted and flightless birds (p = 0.470, 0.111). For Richness all pairwise comparisons had p < 0.05, except for flighted mammals vs flighted and flightless birds (p = 0.361, 0.161). (J) Shannon’s diversity for migratory and non-migratory birds have significantly different alpha diversities (p = 0.017). Richness between migration modes is not significantly different (p = 0.088). (K) Alpha diversity (richness) scaled against body mass for mammals (R2 = 0.450, p < 2.2e-16) and birds (R2 = 0.367, p < 2.2e-16). (L) In non-flighted mammals alpha diversity scales with body mass (R2 = 0.433, p < 2.2e-16) but in flighted mammals (bats) the relationship is insignificant (R2 = 0.0788, p = 0.808). In both flighted (R2 = 0.343, p = 0.018) and non-flighted birds (R2 = 0.334, p < 2.2e-16) alpha diversity scales with body mass albeit less than [file pone.0293895.s001.pdf]

A

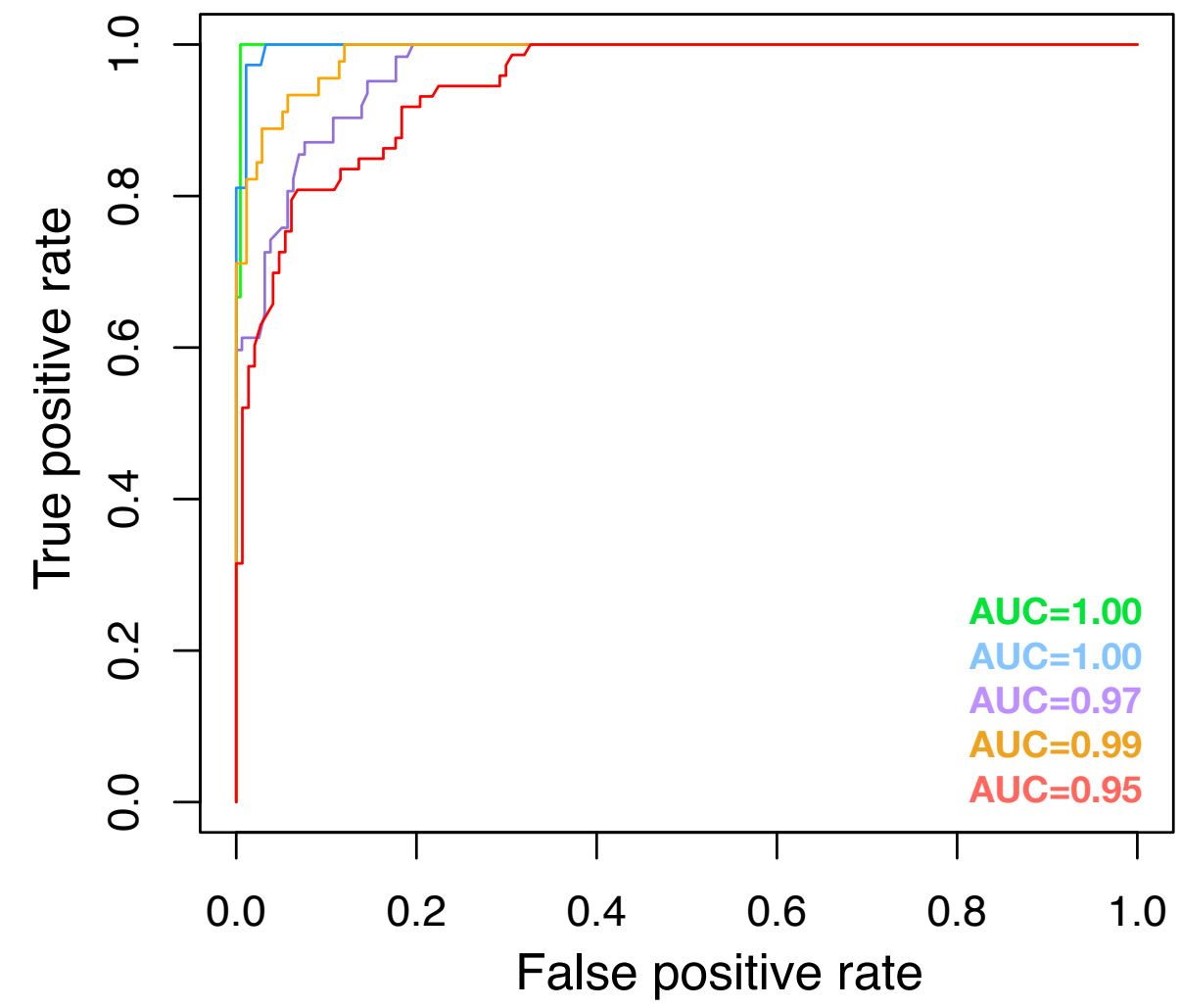

B

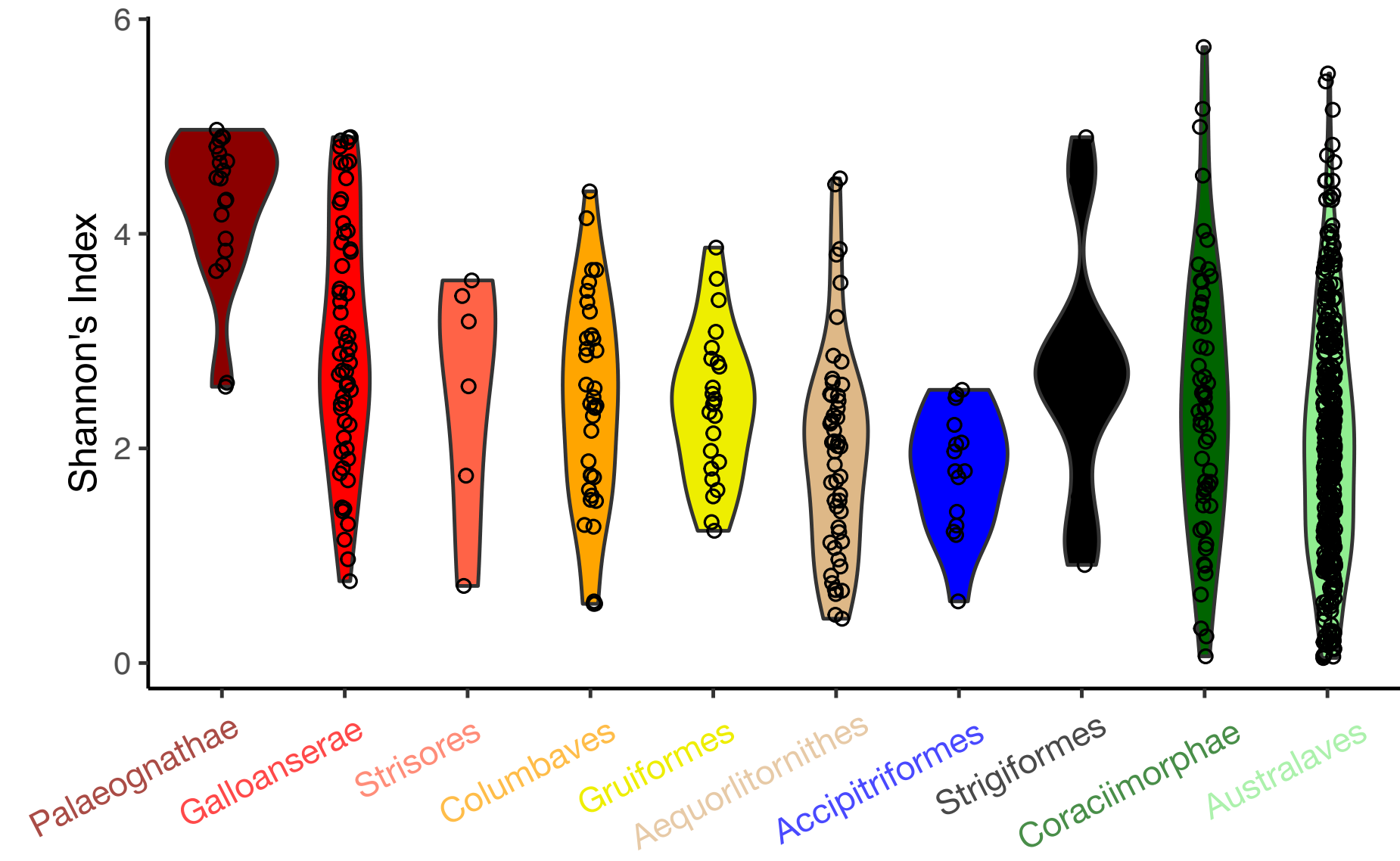

C

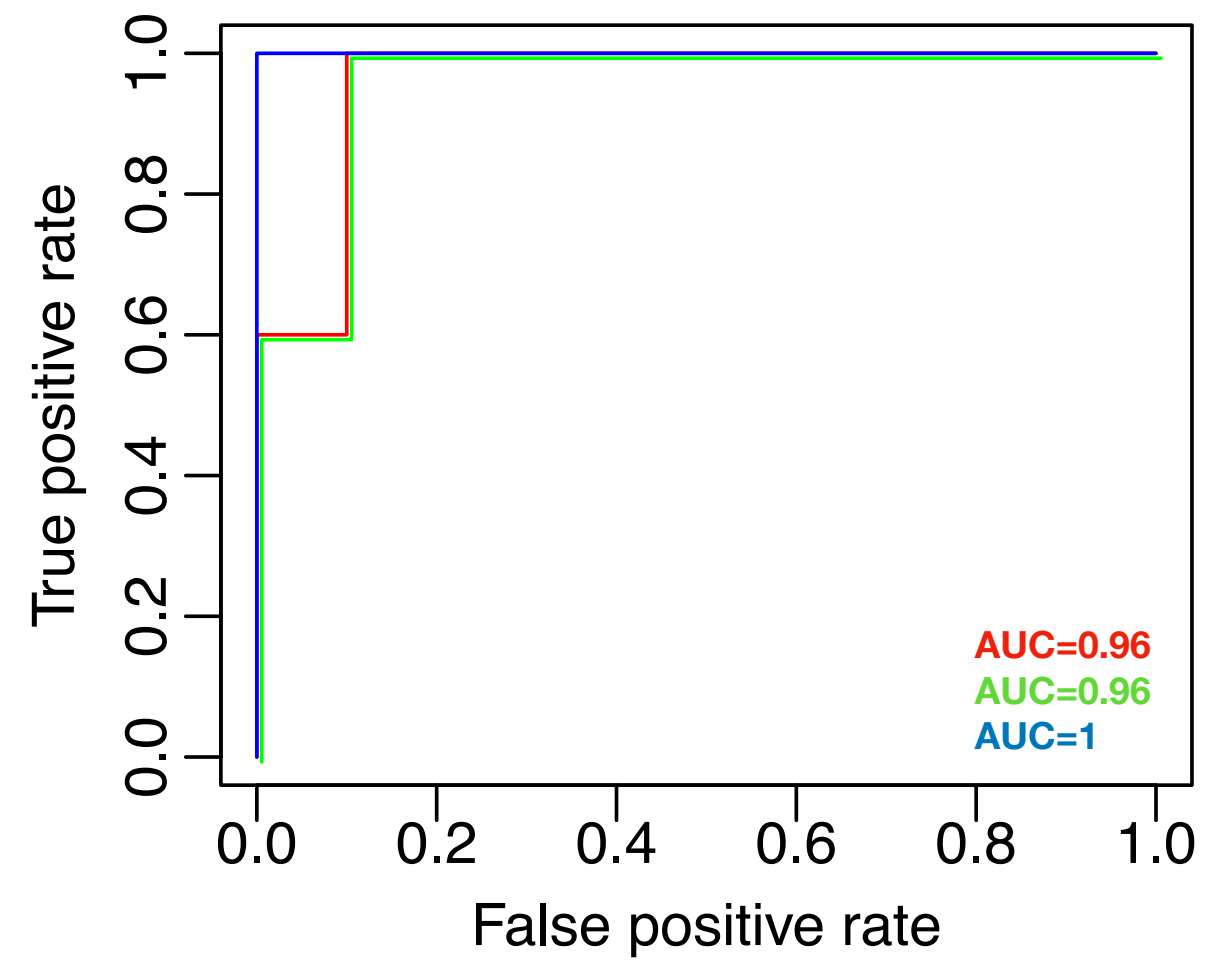

D

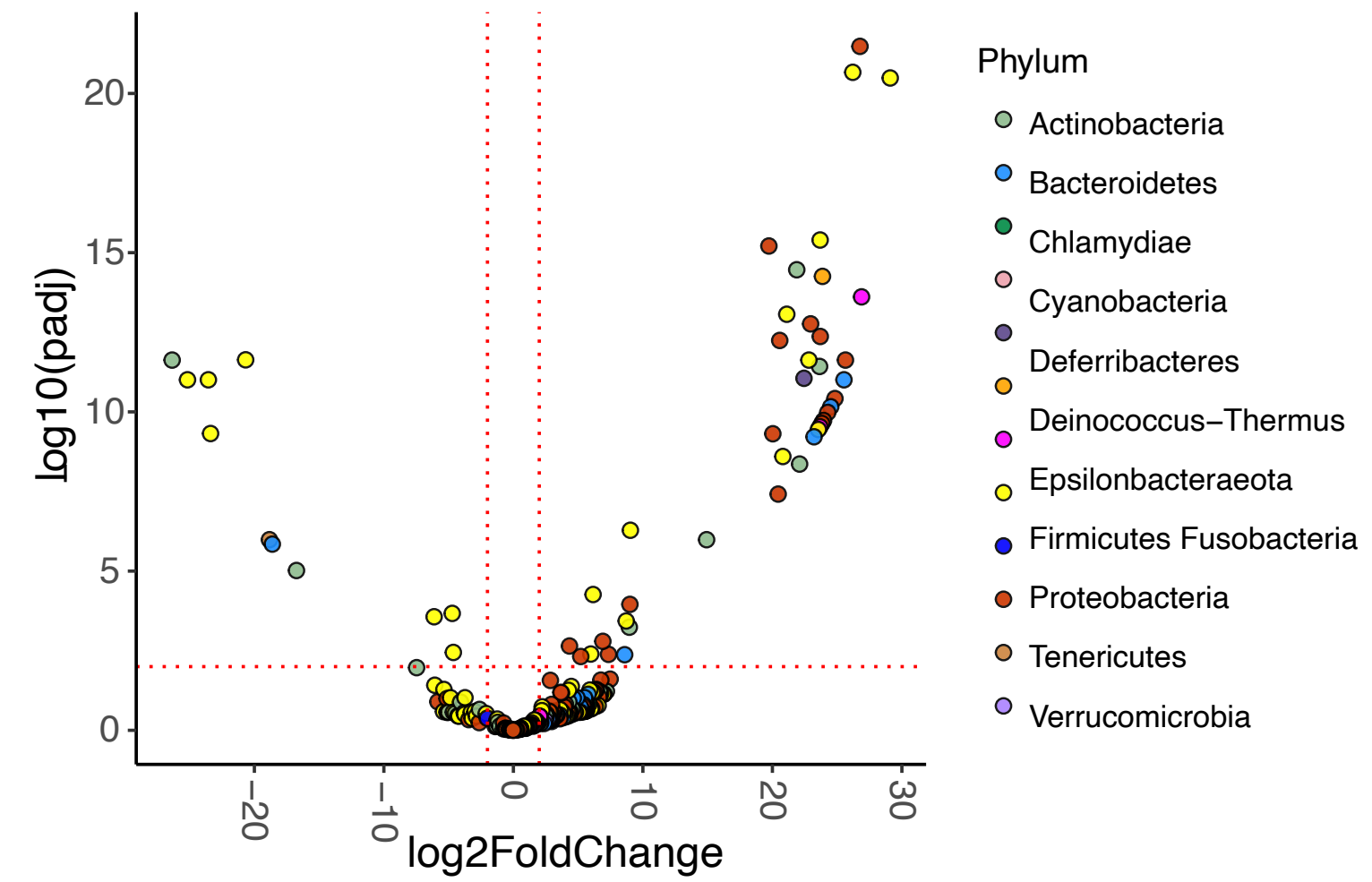

Supplement: S2 Fig — (A) A random forest classifier validates the grouping inferred from the clustering with 19% OOB error and AUCs = 1, 1, 0.97, 0.99 and 0.95. (B) Alpha diversity correlates with phylogeny, with Palaeograthae having the highest (p < 0.05 for all comparisons except with Galloanserae). (C) A random forest classifier supports the beta diversity analysis with 20% OOB error and AUCs = 0.96, 0.96 and 1 for the three dietary groups. (D) DESeq analysis reveals 52 bacterial taxa differently abundant between the dietary groups (log2[fold change] > 2, p < 0.01). (PDF) [file pone.0293895.s002.pdf]

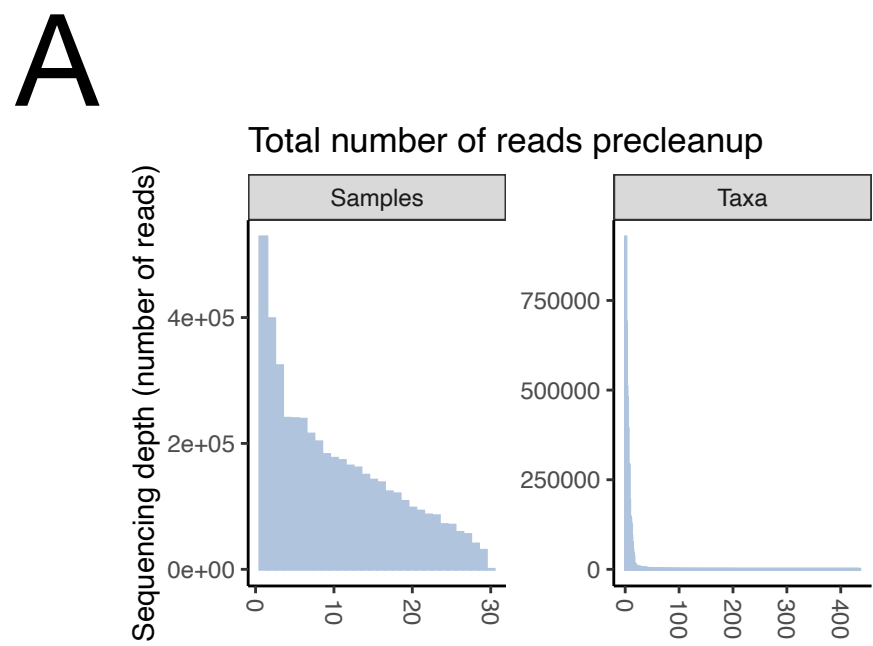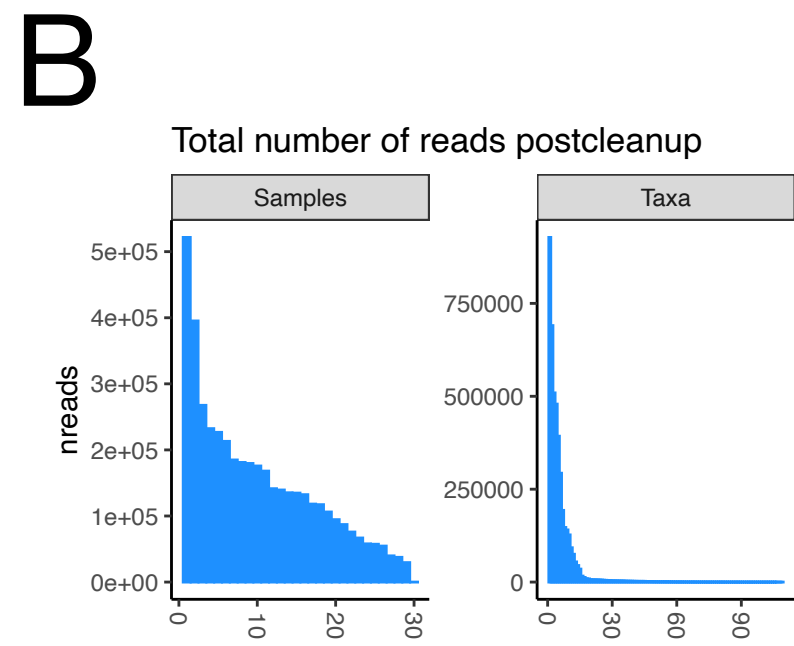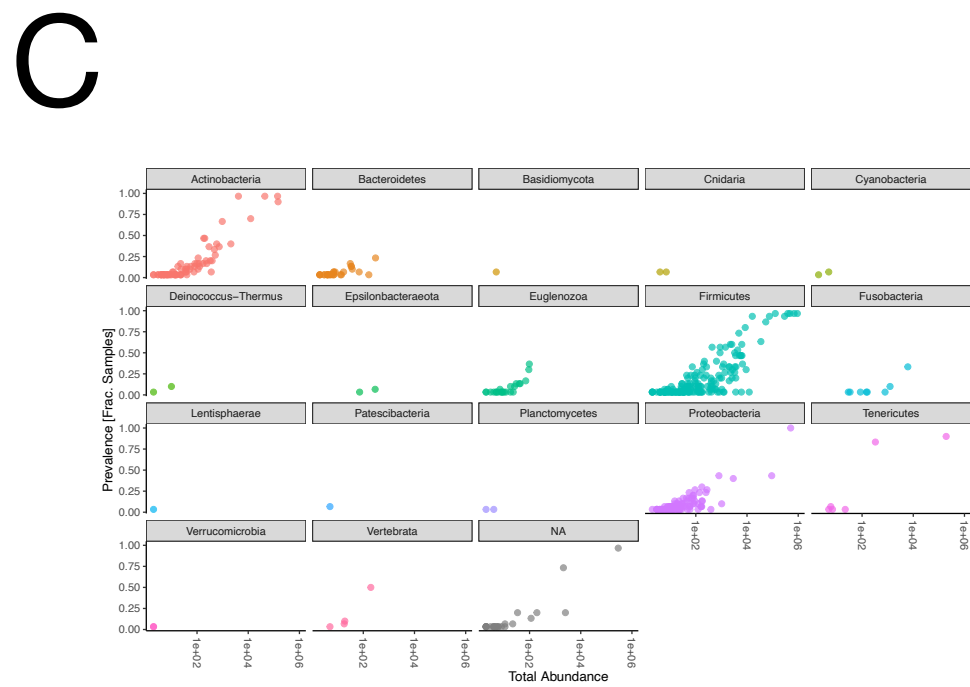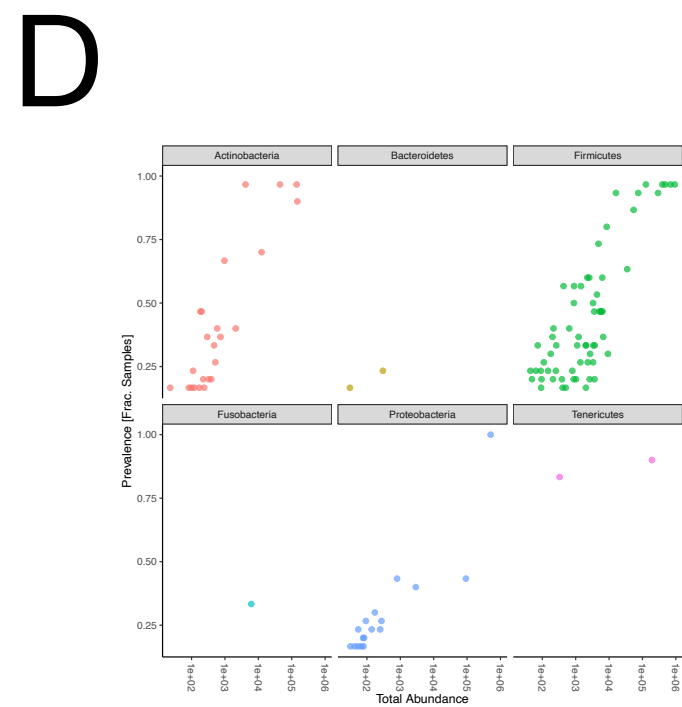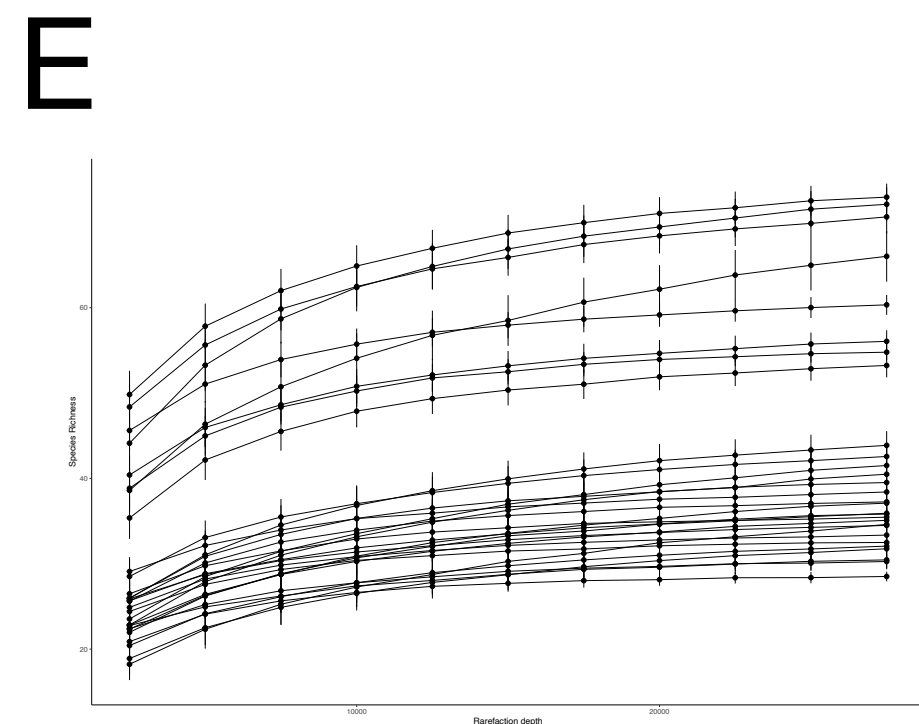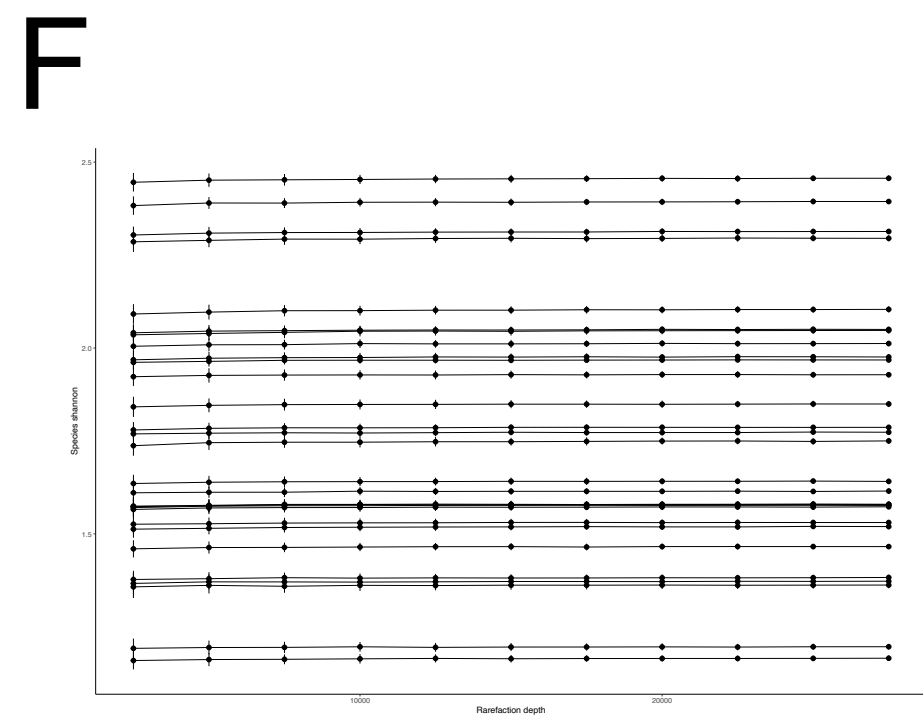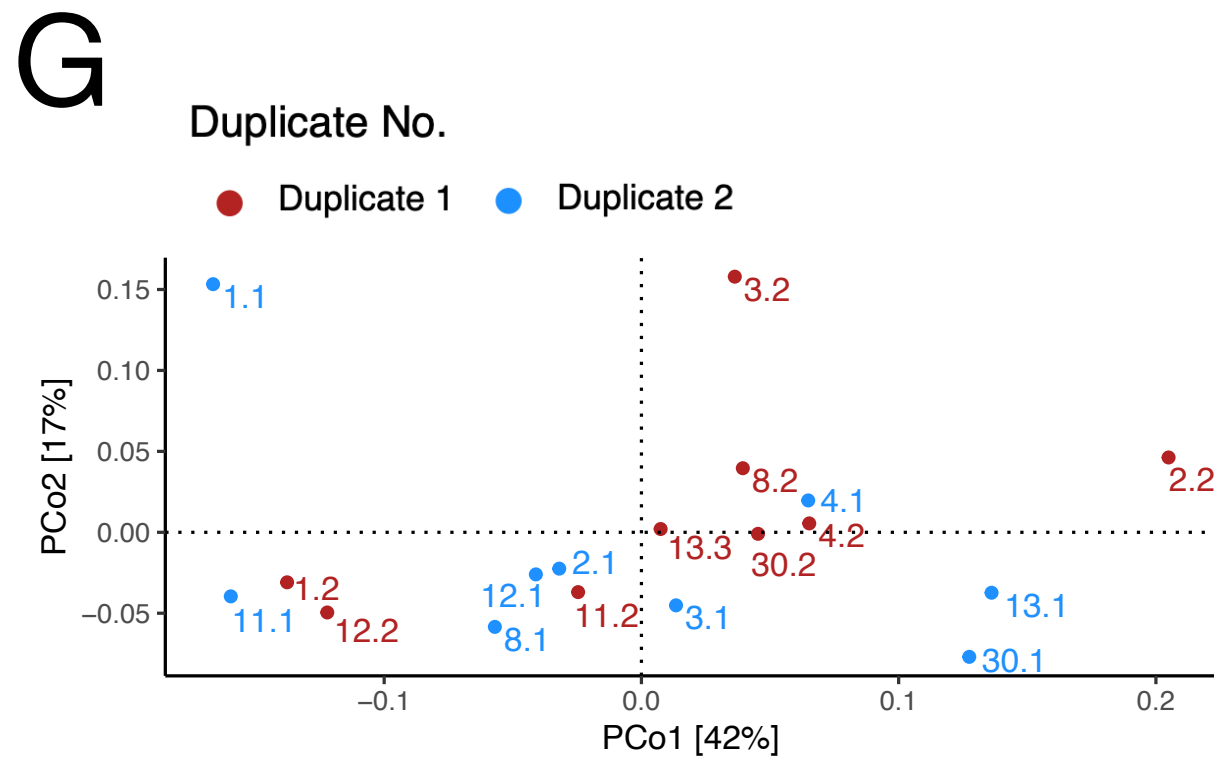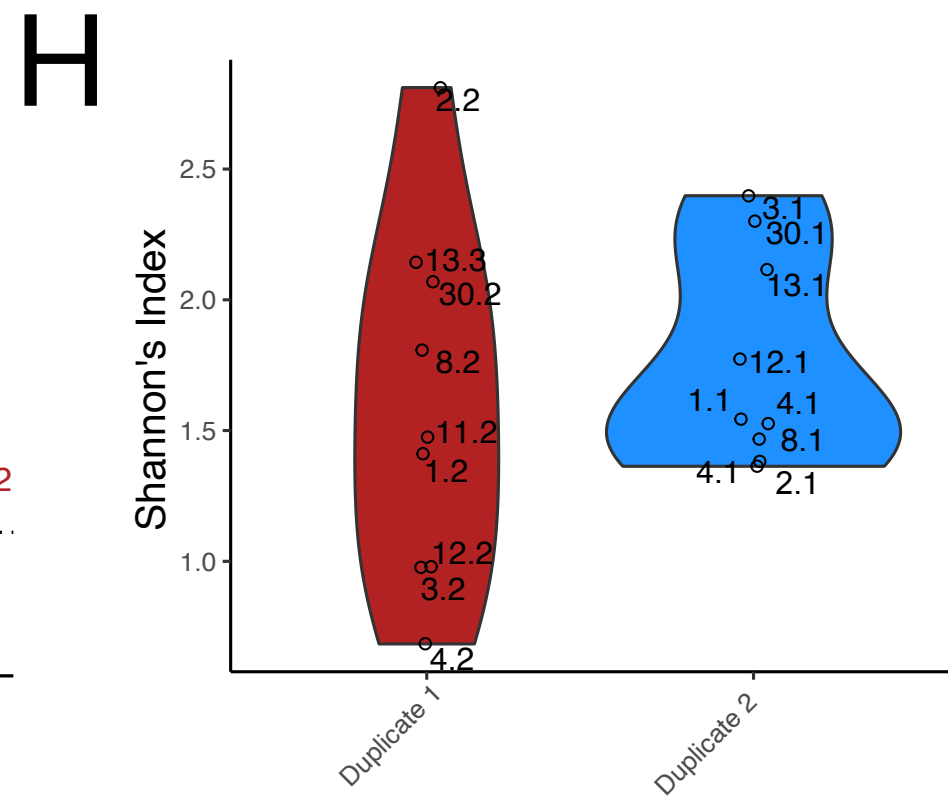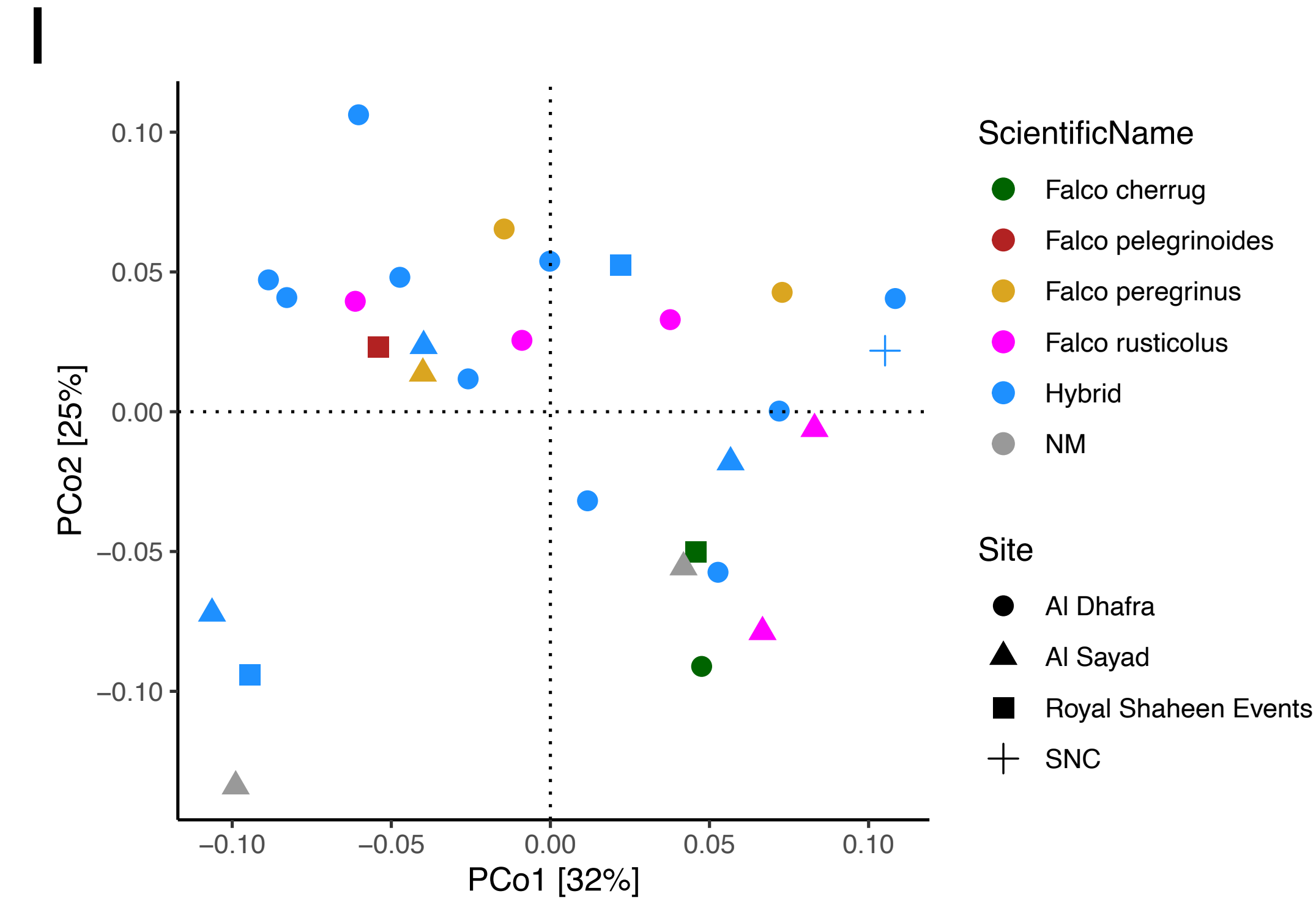

Supplement: S3 Fig — Sequencing depth for each sample and each taxon (A) before filtering any reads, and (B) After removal of lowly abundant ASVs; this does not reduce sequencing depth of either the samples or the taxa significantly. (C) Total abundance and prevalence of phyla in the dataset. (D) After filtering, 6 phyla and a total of 109 taxa remained. One sample was removed on account of low reads, with the resulting phyloseq object having 29 samples. Rarefaction curves for (E) species richness and (F) Shannon’s diversity show plateauing alpha diversity for most samples starting at a rarefaction depth of 15,000 reads. (G) Beta diversity analysis of falcons with duplicates. No marked differences between replicates observed p > 0.05, PERMANOVA. Samples in blue are replicates with higher read counts and samples in red are replicates with lower read counts. (H) Alpha diversity between replicates does not differ markedly as well (p > 0.05 at rarefaction depth = 15,000). (I) PCoA using weighted UniFrac distances of the falcon 16S rRNA gene data reveals separation that is not explained by species (color) or sampling site (shape). (PDF) [file pone.0293895.s003.pdf]
